# Post-Treatment Lyme Disease Syndrome in Six European Countries Using Data from the Burden of Lyme Disease (BOLD) Study

**DOI:** 10.3390/pathogens15080809

**Published:** 2026-08-01

**Authors:** Holly Yu, Amanda R. Mercadante, Kate Halsby, Alexandra Loew-Baselli, Ye Tan, Frederick J. Angulo, Elizabeth Begier, Mendwas Dzingina, Johan S. Berglund, Anna Moniuszko-Malinowska, Viliam Cibik, Dagmar Zakova, Franc Strle, James H. Stark, on behalf of the BOLD Study Group

**Affiliations:** 1 HTA Value and Evidence, Pfizer, Inc., Collegeville, PA 19426, USA; frederick.j.angulo@pfizer.com; 2 HTA Value and Evidence, Pfizer, Inc., New York, NY 10001, USA; amandarmerc@gmail.com; 3 Pfizer Research & Development, Tadworth, Surrey KT20 7NS, UK; kate.halsby@pfizer.com (K.H.); mendwas.dzingina@pfizer.com (M.D.); 4 Pfizer Corporation Austria GmbH, Floridsdorfer Hauptstraße 1, 1210 Vienna, Austria; alexandra.loew-baselli@pfizer.com; 5 Evidence Generation Statistics, Pfizer Inc., Cambridge, MA 02139, USA; ye.tan@pfizer.com (Y.T.); james.h.stark@pfizer.com (J.H.S.); 6 Pfizer Healthcare Ireland Unlimited Company, The Watermarque Building, Ringsend Road, Dublin 4, D04 K7N3 Dublin, Ireland; elizabeth.begier@pfizer.com; 7 Department of Health, Blekinge Institute of Technology, Valhallavägen 1, 371 79 Karlskrona, Sweden; johan.sanmartin.berglund@bth.se; 8 Department of Infectious Diseases and Neuroinfections, Medical University of Białystok, ul. Zurawia 14, Blok D, 15-540 Bialystok, Poland; anna.moniuszko-malinowska@umb.edu.pl; 9 Vseobecna Ambulancia pre Dospelych, Pruské 293, 018 52 Pruské, Slovakia; viliam.cibik@gmail.com; 10 Ambulancia Vnutorneho Lekarstva, 911 01 Trencin, Slovakia; zakova.dagmar@gmail.com; 11 Department of Infectious Diseases, University Medical Centre Ljubljana, Japljeva 2, 1525 Ljubljana, Slovenia; franc.strle@kclj.si

**Keywords:** Lyme borreliosis, Lyme disease, patient reported outcomes, post-treatment Lyme disease syndrome, prevention, quality of life

## Abstract

Up to 10% of patients with Lyme borreliosis (LB) will experience symptoms that fulfill the Infectious Disease Society of America (IDSA) criteria of post-treatment Lyme disease syndrome (PTLDS). This study assesses PTLDS in the Burden of Lyme Disease (BOLD) study through clinical assessment (“PTLDS Assessed by Clinicians”) and by post hoc evaluation of patient-reported outcomes, including the 36-Item Short Form Health Survey, Fatigue Severity Scale, and Cognitive Failures Questionnaire (“PTLDS Assessed by Questionnaire”). Among 242 LB cases who completed antibiotic treatment and had objective manifestations stabilized or resolved, 99 (40.9%) reported ≥1 symptom of fatigue, musculoskeletal pain, or cognitive impairment 10 months after diagnosis. Fifteen (6.2%) cases met the PTLDS criteria assessed by either clinicians or predefined questionnaire severity thresholds; four (1.7%) met both. PTLDS cases assessed by questionnaires and LB cases with persistent symptoms both reported greater symptom severity and poorer physical functioning than PTLDS cases assessed by clinicians. These exploratory findings suggest that clinician- and questionnaire-based assessments may identify partially different subsets of patients with PTLDS and either method could underestimate the proportion of patients with PTLDS.

## 1. Introduction

Lyme borreliosis (LB) is the most common tick-borne infectious disease in Europe. Between 5% and 10% of patients with LB will experience prolonged (or recurrent) symptoms lasting ≥6 months that fulfill the criteria of post-treatment Lyme disease syndrome (PTLDS) [1,2,3,4]. The most cited case definition for PTLDS was proposed in the 2006 clinical practice guidelines by the Infectious Disease Society of America (IDSA) [5]. These criteria define PTLDS as clinician-diagnosed LB with resolution or stabilization of the objective disease manifestations after antibiotic treatment; significant fatigue, widespread musculoskeletal pain, or cognitive difficulties occurring within 6 months of diagnosis that last for ≥6 months after the end of treatment and cause substantial reduction in activities; other potential causes for the symptoms must be excluded [5]. Although alternative frameworks have been proposed by other expert groups and national authorities [6,7], the 2006 IDSA definition remains among the most stringent and rigorously specified PTLDS definitions. Compared with other available frameworks, the 2006 IDSA definition provides more explicit criteria regarding symptom persistence, functional impairment, temporal association with treated LB, and exclusion of alternative explanations, facilitating transparent and reproducible application in research settings. Consequently, the 2006 IDSA was selected as the conceptual framework for the present exploratory analysis.

Previous clinical studies of PTLDS have utilized patient-reported outcomes (PROs) assessed through validated questionnaires to provide metrics for the frequency, severity, and health-related quality of life (HRQoL) impact on patients who continue to experience symptoms for at least 6 months following antibiotic treatment [8]. LymeProspect used the Checklist Individual Strength (CIS), Cognitive Failures Questionnaire (CFQ), and the 36-Item Short Form Health Survey (SF-36) to measure the severity of fatigue, cognitive impairment, and pain, respectively, and the prevalence of these symptoms in LB patients compared to controls [4]. Results showed the presence of persistent symptoms, without meeting the PTLDS physical activity reduction criterion. The study also found an association between the existence of persistent symptoms and LB; 27.2% of LB patients experienced persistent symptoms, significantly higher than the 21.2% in the general population cohort and 23.3% in the tick-bite without LB cohort, implying that 3.9% to 6.0% of persistent symptoms are attributable to LB.

The Burden of Lyme Disease (BOLD) study was a prospective epidemiological study conducted across six European countries [9]. Preliminary results from the BOLD study estimated an overall LB incidence of 656.5 per 100,000 population in 2021 and 660.6 per 100,000 population in 2022 [10]. Over 70% of LB cases were diagnosed as solitary erythema migrans; among disseminated cases, arthritis predominated, and Slovakia observed the highest proportion of Lyme arthritis cases (71%) [11]. BOLD also collected data on the PTLDS burden of LB. Several analyses from the BOLD study have been published previously, including estimates of the incidence of LB, clinical manifestations and symptoms, and patient-reported HRQoL and symptom severity [9,10,12]. Here, we provide an exploratory assessment of the proportions and characteristics of PTLDS (either collected from clinical assessments or through meeting score-based criteria from PRO instruments) among LB cases enrolled in the study and descriptively compare the impact of nonspecific pain, fatigue, and cognitive impairment between the LB cases that qualify as IDSA PTLDS and those with persistent symptoms that do not fulfill the reduction in physical activity PTLDS case criterion.

## 2. Materials and Methods

### 2.1. Study Design and Population

The BOLD study protocol was previously published [9]. Consenting members of any age from 14 participating general/primary care practices in high-incidence regions of Europe with suspected LB were enrolled between April 2021 and July 2022 and were assessed in up to five study visits over 2 years following diagnosis. While the BOLD methodology paper counted three sites separately according to Polish law, for the purpose of this analysis two were combined, as one site was the referral site of the other. Local ethics committees for each participating site approved this study (Appendix B Table A1).

Medically attended LB cases enrolled at Visit 1, which often coincided with the primary care visit where the case was newly diagnosed with, or suspected of having, LB. Visit 2 took place the following month, capturing the acute phase of illness. All LB cases were invited to return for Visit 3 (10–11 months after diagnosis) and were evaluated for PTLDS in two ways, (i) through clinical assessment of PTLDS by the clinician at each study site using pre-set criteria (“PTLDS assessed by clinicians”), and (ii) by post hoc evaluation of their ongoing symptoms combined with PRO data collected through self-completed/assisted standardized questionnaires (“PTLDS assessed by questionnaires”). Both definitions considered the IDSA criteria for identifying PTLDS, with the only difference being the approach used to evaluate symptom severity and HRQoL impact. Detailed criteria for these two subgroups are described in Table 1. After Visit 3, cases who reported ongoing or new onset of symptoms related to their LB diagnosis, confirmed through medical records and patient interview, were invited to attend Visit 4 (approximately 16–18 months after diagnosis), and those experiencing ongoing symptoms after Visit 4 were invited back for Visit 5 (22–24 months after diagnosis). Patients could satisfy both PTLDS definitions, but it was not necessary to meet both to have a classification of PTLDS as operationalized herein.

The presence and severity of PTLDS-associated symptoms as operationalized herein (pain, fatigue, and cognitive function) were assessed at approximately months 10, 18, and 24. The PRO instruments used included SF-36 (measuring bodily pain and physical functioning through the corresponding SF-36 subscales), Fatigue Severity Scale (FSS; measuring fatigue), and CFQ (measuring cognitive impairment). Higher scores on the SF-36 indicate better health status [13], and scores ≤55 reflected significant impairment. In contrast, higher scores on the FSS (≥4) and on the CFQ (≥44) indicated severe fatigue and more frequent cognitive failures, respectively. The cut-offs for determining severity in each questionnaire were chosen for alignment of the PTLDS evaluation with a previous European LB trial [14], which applied predefined thresholds derived from population norms (rather than thresholds derived from the BOLD study population). Cases completed the SF-36 at either Visit 1 or Visit 2, and then at all subsequent visits. The FSS was completed at all visits, and the CFQ was completed at Visits 3–5. Only PRO measurements from cases with reported symptoms lasting ≥6 months and who attended Visit 3 and completed the full questionnaire (answered all items) were included in the PTLDS calculations.

Each enrolled LB case was age- (±5 years) and site-matched with a control who did not have LB. Eligibility criteria for controls were being a member of a participating patient practice at the time of enrolment of a diagnosed LB case, providing informed consent, and not having active LB in the last 90 days [9]. Control participants completed Contact 1 at enrollment and Contact 2 approximately 16–18 months later, at which time health survey information was collected.

### 2.2. Analysis

Patient demographic characteristics and disease manifestations were collected up to Visit 3. Localized manifestation required an erythema migrans rash, whereas disseminated manifestations could include Lyme neuroborreliosis, Lyme arthritis, Lyme carditis, ocular LB, or LB diagnosis without established LB clinical manifestations as defined by the BOLD study protocol [9] (e.g., systemic symptoms only). Comorbidities were captured at Visit 3 using the Charlson Comorbidity Index (CCI) score. Clinician-reported antibiotic utilization was collected at all visits. Cases with a new diagnosis of LB during study follow-up were excluded from further analysis. Data were summarized with counts (*n*), percentages (%), means (standard deviation), or medians (interquartile range). Associated 95% confidence intervals (CI) for the percentages were calculated using the Clopper–Pearson method. All descriptive statistics were generated in SAS version 9.4 (SAS Institute, Cary, NC, USA).

**Figure 1:**
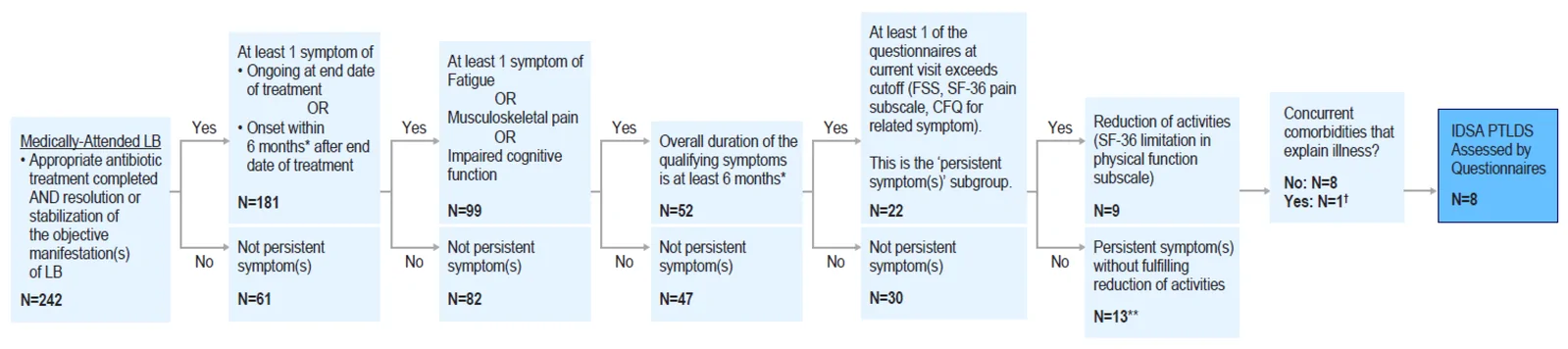
Flowchart for identifying the IDSA PTLDS by questionnaires subgroup. * 6 months = 180 days; ** 1 participant had comorbidities that could explain their reported symptoms. ^†^ Patients with comorbidities explaining their illness were removed from the analysis. Abbreviations: CFQ = Cognitive Failures Questionnaire; FSS = Fatigue Severity Scale; IDSA = Infectious Disease Society of America; LB = Lyme borreliosis; PTLDS = post-treatment Lyme disease syndrome; SF-36 = 36-Item Short Form Health Survey.

The criteria for identifying cases of “PTLDS assessed by questionnaires” were applied in a stepwise approach at Visits 3 to 5 (Figure 1).

First, medically attended LB cases who completed treatment with an appropriate antibiotic regimen were evaluated for resolution or stabilization of objective manifestations of LB. Second, cases were evaluated for the presence of at least 1 symptom that was ongoing after treatment ended or was newly developed within 6 months of the last day of treatment. Duration was calculated as the earliest start date (or end date of treatment for symptoms ongoing at that timepoint) to the latest end date (or ongoing) plus 1 day for any of these symptoms. Third, the presenting symptoms were limited to either fatigue, cognitive impairment, or musculoskeletal pain, which included neck pain, arthralgias, myalgias, diffuse musculoskeletal pain, and joint stiffness. Fourth, LB cases with a qualifying symptom had to have had the symptom for ≥6 months. Fifth, cases were further required to have reported impaired PRO scores, exceeding the cutoff for at least 1 questionnaire assessing fatigue, pain, or cognitive functioning. Patients meeting this criterion were classified as having ‘persistent symptom(s)’. Sixth, cases were then evaluated for reductions in physical activities according to the SF-36 physical function subscale. Those with persistent symptoms who did not exhibit a reduction in physical activities were categorized as having ‘persistent symptoms without fulfilling reduction in physical activities’ (i.e., not meeting the IDSA PTLDS activity reduction requirement). In the last step, cases with persistent symptoms who also had an impaired physical functioning score exceeding the SF-36 cutoff and no concurrent comorbidities (as assessed by physicians based on medical records and patient interviews) that could account for their illness were classified as fulfilling the IDSA PTLDS criteria.

## 3. Results

### 3.1. Patient Summary

The initial assessment of PTLDS (as operationalized herein) occurred at Visit 3, approximately 10–11 months after diagnosis, for which 251 LB cases completed the visit. Of these, 242 LB cases had completed their antibiotic regimen with resolution or stabilization of LB symptoms and were eligible for the PTLDS analysis (Figure 1). Cases had a mean age of 55.7 years, 55.8% were female, and 64.9% had erythema migrans (Table 2). Cases also had a low CCI score (2.8), suggesting mostly mild to moderate severity of comorbid conditions.

At 10 months, one or more ongoing symptoms were reported by 181 LB cases, of which 99 patients reported at least one symptom of fatigue, musculoskeletal pain, or cognitive impairment. Of these 99 patients, 52 had a qualifying symptom(s) that lasted for ≥6 months; 22 of these had persistent symptoms with at least one PRO outcome that exceeded the cut-off, including 8 who fulfilled the PTLDS criteria as operationalized herein (Figure 1). These patients with persistent symptoms had a mean age of 59.6 years, 59.1% were female, 72.7% had a disseminated manifestation (54.5% Lyme arthritis), and a mean CCI score of 3.5, suggesting moderate severity of comorbid conditions (Table 2).

Across all visits, a total of 8 LB cases were identified that met the criteria for “PTLDS assessed by questionnaires”, while 11 cases (10 at Visit 3 and 1 additional case at Visit 4) were identified with the “PTLDS assessed by clinicians” definition (Appendix C Table A2). Of the 242 LB cases with resolution or stabilization of LB symptoms after antibiotic treatment, 15 (6.2%) cases met at least one of the two PTLDS definitions, and 4 (1.7%) met both. Among the 15 LB cases that satisfied either PTLDS definition, 4 were diagnosed with localized LB and 11 with disseminated LB. The proportion of patients classified as having PTLDS was numerically lower in patients with localized LB than in those with disseminated disease: out of 157 cases of solitary erythema migrans, 1 (0.6%) fulfilled the criteria for PTLDS assessed by questionnaires and 4 (2.5%) as assessed by clinical workup, while the corresponding proportions for disseminated LB were both 7/85 (8.2%), respectively (Table 2). Within the PTLDS assessed by questionnaires subgroup (*n* = 8), patients were, on average, male (62.5%), older (mean age of 60.4 years), and had comorbidities of moderate severity (mean CCI score of 3.9). They all had Lyme arthritis manifestations, with the exception of one case with a localized infection. LB cases with PTLDS assessed by clinicians had a higher proportion of males (63.6%), were younger (mean age of 50.8 years), had the lowest CCI scores (mean CCI score of 2.1), and most (63.6%) had disseminated infections. The majority (54.5–87.5%) of all PTLDS cases, by either definition, were enrolled from Slovakia.

Table 3 presents antibiotic use for the treatment of LB stratified by subgroup. The persistent symptoms subgroup and both PTLDS subgroups reported a numerically higher mean total number of days using antibiotics than the enrolled and treated LB cases (15.1 days for LB cases, 19.3 days for persistent symptoms cases, 20.0 days for PTLDS cases assessed by questionnaires, and 21.5 days for PTLDS cases assessed by clinicians). Patients in the persistent symptoms and PTLDS (as operationalized herein) subgroups had a numerically higher proportion using two or more different antibiotics and a higher proportion with delayed treatment initiation (>30 days after the first documented symptom) than LB cases.

### 3.2. LB Cases Meeting PTLDS Criteria

Figure 1 shows the application of the criteria for PTLDS assessed by questionnaires to the 242 eligible LB cases that had resolution or stabilization of their objective LB manifestations after completing an appropriate antibiotic regimen. Among the 242 LB cases, 181 (74.8%) reported at least one ongoing or new symptom after treatment. Approximately 41% (99/242) LB cases reported at least one symptom of fatigue, musculoskeletal pain, or cognitive impairment. A subset of these 99 cases (52/99, 21.5%) had qualifying symptoms that lasted for ≥6 months after treatment completion. Among these cases, 22 (9.1%) exceeded the questionnaire cutoffs for symptom severity based on the FSS, SF-36 pain subscale, or CFQ. This subset of patients was designated as having ‘persistent symptom(s)’. Nine cases (3.7%) additionally demonstrated reduction in physical functioning as measured by the SF-36 physical function subscale, thereby fulfilling the activity-limitation criterion of IDSA PTLDS. Among these nine, 1 case had comorbidities that could explain the symptoms, resulting in 8 cases (3.3%) meeting the IDSA-defined PTLDS criteria by questionnaires. All 8 PTLDS cases (100%) assessed by questionnaires reported severe bodily pain, fatigue, and physical impairment and could be defined by the pain, fatigue, and physical functioning PROs alone, while a lower proportion would have been identified based on severe impairment in cognitive function (3/8, 37.5%) (Appendix C Table A3).

There were 11 cases over Visits 3–5 that had PTLDS when assessed by clinicians, of which 63.6% met the SF-36 bodily pain subscale cut-off for severe pain, 63.6% met the FSS cut-off for severe fatigue, 18.2% met the CFQ cut-off for severe cognitive impairment, and only 36.4% would have satisfied the clinically relevant thresholds for the SF-36 physical function subscale for severely disabled function (Appendix C Table A3).

Eight LB cases with persistent symptoms would ultimately be categorized as PTLDS assessed by questionnaires (Figure 1), and 11 patients with persistent symptoms would be categorized as PTLDS assessed by clinicians.

### 3.3. LB Cases with Persistent Symptom(s) Without Fulfilling Reduction in Physical Activities PTLDS Criterion

**Figure 2:**
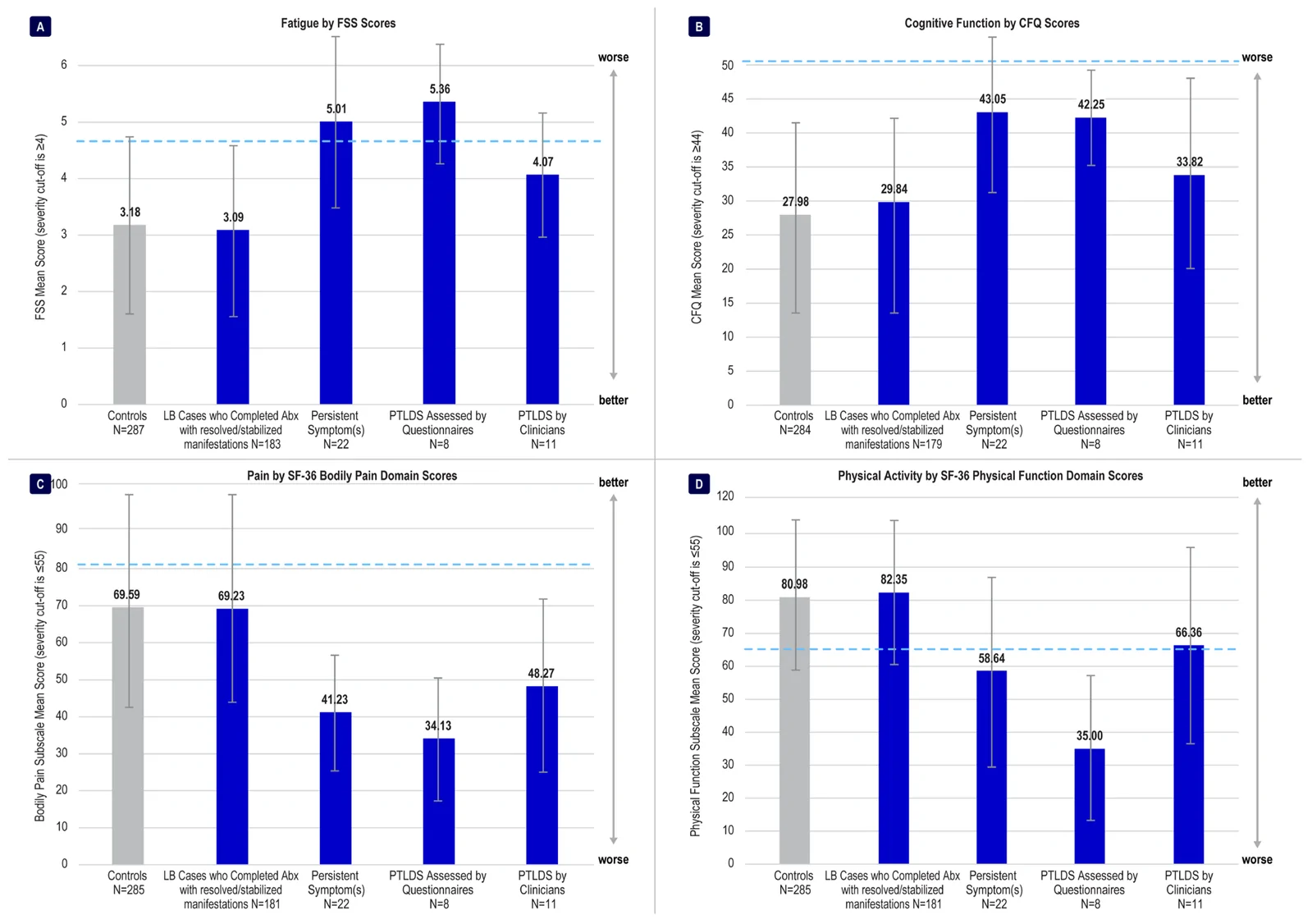
Scores measured by (**A**) FSS, (**B**) CFQ, (**C**) SF-36 Bodily Pain Subscale, and (**D**) SF-36 Physical Function Subscale by subgroup. Dashed lines indicate the severity cut-offs for each questionnaire. Note: The subgroups displayed are not mutually exclusive. The persistent symptoms subgroup includes all questionnaire-defined PTLDS cases, and four patients satisfied both the questionnaire-defined and clinician-defined PTLDS classifications. The figure is intended to descriptively display questionnaire score distributions across study classifications and should not be interpreted as a comparison of independent patient groups. Controls were assessed at Contact 2 (approximately 16–18 months after enrollment), whereas the primary PTLDS assessment for LB cases occurred at Visit 3 (approximately 10–11 months after diagnosis). Controls were not used to define PTLDS status or questionnaire thresholds. Control data are presented for contextual information only and should not be interpreted as providing an estimate of symptom burden attributable to LB. Abbreviations: Abx = antibiotics; CFQ = Cognitive Failures Questionnaire; FSS = Fatigue Severity Scale; LB = Lyme borreliosis; PTLDS = post-treatment Lyme disease syndrome; SF-36 = 36-Item Short Form Health Survey.

At month 10, 22/52 (42.3%) had symptom-specific PRO scores above the severity cut-off, meeting the criteria for persistent symptoms, of whom 13 (25.0%) did not fulfill the reduction in physical activities criterion. The persistent symptoms subgroup was not mutually exclusive from the PTLDS subgroups; all questionnaire-defined PTLDS cases were included within the persistent symptoms classification, and 4 satisfied both questionnaire-defined and clinician-defined PTLDS definitions. Twenty persistent symptom cases (90.9%) reported severe bodily pain (as measured by the SF-36 bodily pain subscale), while fewer patients exceeded the PRO cut-offs for CFQ (9/22, 40.9%), FSS (18/22, 81.8%), and SF-36 physical function subscale (9/22, 40.9%) (Figure 2; Appendix C Table A3).

The IDSA criteria only consider pain, cognitive difficulties, and fatigue (Table 1); however, of 242 LB cases that completed an appropriate antibiotic regimen with resolution or stabilization of LB manifestations, 82 (34%) reported a symptom other than fatigue, musculoskeletal pain, or cognitive impairment at the end of treatment. Based on the IDSA-defined criteria for PTLDS, 13/242 (5.4%) treated LB cases reported an ongoing (≥6 months post-treatment) fatigue, pain, or cognition symptom that exceeded the severity cut-offs; however, they did not meet the physical functioning impairment cut-off and did not qualify as PTLDS (as operationalized herein). Among these 13 cases, one had a comorbidity that could explain the symptoms (Figure 1).

## 4. Discussion

This study provides a descriptive characterization of PTLDS in a European LB cohort by applying the IDSA framework using both questionnaire-based and clinician-based assessment approaches. Although both approaches were derived from the same conceptual definition, they identified different patient populations: while 6.2% (15/242) of LB cases met at least one PTLDS definition, only 1.7% (4/242) met both, highlighting potential challenges in operationalizing PTLDS criteria across clinical and research settings.

One explanation for the observed discordance is that persistent symptoms after LB are inherently subjective and may be characterized differently depending on the assessment method used. While PROs are inherently subjective, standardized PRO instruments provide a reproducible method for measuring symptom burden and functional impact, whereas clinician assessments may vary according to questioning, interpretation, and documentation practices. By definition, questionnaire-defined PTLDS cases met predefined thresholds for fatigue, pain, and physical functioning impairment, whereas a lower proportion of clinician-defined PTLDS cases satisfied those same questionnaire thresholds. These differences reflect variation in symptom attribution, comorbidity assessment, functional impairment thresholds, subgroup heterogeneity, or measurement differences between assessment approaches.

The discrepancy between assessment approaches may also partly reflect the emphasis placed by the IDSA framework on substantial reductions in physical functioning. Patients with persistent symptoms who did not meet the physical functioning criterion nevertheless reported impairments in HRQoL and symptom-specific measures. Consistent with this observation, only 36.4% of clinician-defined PTLDS cases met the SF-36 physical functioning severity threshold. These exploratory findings suggest that clinicians may place greater emphasis on overall symptom burden rather than physical functioning limitations alone.

The prevalence of questionnaire-defined PTLDS in BOLD (8/242, 3.3%) was lower than previously reported estimates, which range from 13.7% to 38% [1,4,15,16]. Differences likely reflect variation in follow-up duration, approaches to symptom ascertainment and reporting, interpretation of the IDSA case definition, choice of PRO instruments, and differences in the criteria used to define and enroll LB cases across studies. Notably, BOLD required documentation of persistent symptoms for ≥6 months through patient interviews, clinician assessment, and medical record review, before applying the PRO instruments and physical limitations criteria. In contrast, studies such as LymeProspect [14] identified persistent symptoms directly from questionnaires and duration criteria rather than through a separate symptom ascertainment step prior to questionnaire assessment. In addition, LymeProspect did not require an additional physical functioning impairment threshold as part of the case identification [14]. These methodological differences substantially influence case ascertainment and may partly explain the broad range of PTLDS prevalence estimates reported in the literature. The frequency, clinical characteristics, and estimated burden of PTLDS are inherently dependent on the case definition applied, highlighting the importance of considering diagnostic criteria when interpreting and comparing findings across studies and underscoring the need for standardization of PTLDS definitions and assessment approaches.

Consistent with this interpretation, we identified a subset of patients (13/242, 5.4%) with persistent symptoms and reduced HRQoL who did not meet all PTLDS criteria, most commonly because they lacked substantial impairment in physical functioning. These symptoms might have been classified as PTLDS under broader definitions used in other studies [17]. Together with the observation that approximately one-third of LB cases reported persistent symptoms outside the core IDSA domains, these exploratory findings suggest that many patients continue to experience symptoms following LB treatment, regardless of the PTLDS definition applied.

The PRO instruments and the cut-offs used in this study were selected to align with LymeProspect and represent commonly used, validated measures of fatigue, pain, cognitive difficulties, and HRQoL. However, these instruments have not been specifically psychometrically tested in LB populations, a limitation that extends across much of the PTLDS literature. LB-specific instruments such as the 36-item post-Lyme questionnaire of symptoms (PLQS) and the General Symptom Questionnaire-30 (GSQ-30) were developed based on PTLDS clinical and research experience and have been psychometrically tested in LB patients [18,19,20], but they were not assessed for use in this study at the time of design and may be valuable tools for future studies.

An important limitation of this study is the small number of PTLDS cases per subgroup, which limits interpretability of the subgroup results. It was also not possible to draw significant conclusions about PTLDS over time, as only patients with ongoing symptoms were invited back for additional assessment, further reducing case numbers. A second limitation relates to the control group included in the BOLD study. Health survey information for controls was collected at a different time point than the primary PTLDS assessment for LB cases, and the selection criteria did not preclude a remote history of LB (beyond the most recent 90 days). Although controls were not used to define PTLDS status or questionnaire thresholds, these factors limit the extent to which control data can be used to contextualize symptom burden observed in the LB cohort. Accordingly, the control data were intended to provide contextual information only and should not be interpreted as a direct comparison group for evaluating the validity of the PTLDS classifications. Further, among LB cases, symptom reports were based on symptoms that were new or had worsened following treatment; as such, these exploratory findings describe post-treatment symptom burden but should not be interpreted as establishing causality. Of note, patient-reported HRQoL, fatigue, pain, and cognitive function in LB cases and matched controls from the BOLD study have been published elsewhere [12]. Finally, most patients with PTLDS (as operationalized herein) were enrolled from Slovakia, limiting the generalizability of these PTLDS patient characteristics to other regions.

A strength of this study is the robust BOLD study methods. The BOLD study incorporated medical record validation to ascertain symptoms and up to two years of follow-up after LB diagnosis, exceeding the duration of previous European prospective PTLDS studies [4,21]. Moreover, the unique ability to descriptively compare two methods of PTLDS assessment within one study and have multiple visits and subgroup data allowed for greater breadth of knowledge about these LB patients and their symptoms.

The exploratory findings of this study provide insight into how current PTLDS definitions function in both clinical and research settings. The limited overlap between clinician-assessed and questionnaire-assessed PTLDS may suggest that the two assessment approaches identify partially different groups of patients experiencing ongoing symptoms after treatment for LB. Standardized assessment strategies that incorporate both clinician evaluation and validated patient-reported outcomes may provide a more comprehensive characterization of post-treatment symptoms and their impact on daily functioning. Future studies should seek to establish consensus definitions, validate PTLDS-specific outcome measures, and determine which symptom domains and functional impairments are most clinically meaningful. Greater harmonization of PTLDS assessment methods would improve comparability across studies and facilitate a clearer understanding of the epidemiology and burden of persistent symptoms following LB.

## 5. Conclusions

This work describes the occurrence of persistent symptoms reported following treatment for LB and underscores the importance of incorporating the patient perspective when assessing PTLDS. Although both of the approaches to assess PTLDS used herein are based on the same case definition, they did not identify the same group of LB patients due to different methods of application (clinical observation versus standardized questionnaires). In general, the different applications of PTLDS criteria across studies hinder comparability and highlights the need for standardized approaches to improve consistency in research and clinical practice. These exploratory study results suggest that strict application of IDSA PTLDS criteria may not capture all patients experiencing persistent post-treatment symptoms.

## Figures and Tables

**Table 1:** Case criteria ^a^ for PTLDS subgroups.

2006 IDSA PTLDS Case Definition	Criteria for PTLDS Assessed by Clinicians	Criteria for PTLDS Assessed by Questionnaires
LB Diagnosis	Diagnosis of LB (clinically [erythema migrans cases only] or laboratory-confirmed)	
Antibiotic Treatment and Stabilization	Completed treatment with an appropriate antibiotic regimen with resolution or stabilization of objective manifestations of LB.	
Symptom Onset and Duration	Onset within 6 months and persists for ≥6 months after completion of antibiotic therapy.	
Symptoms Fatigue; OR Generalized musculoskeletal pain; OR Cognitive difficulties Severity resulting in substantial reduction in activities.	Assessed based on patient interview and medical records for debilitating symptoms (resulting in substantial reduction in activities) of: Fatigue; OR Generalized musculoskeletal pain; OR Cognitive difficulties.	Assessed by patient-reported questionnaires with pre-specified cutoffs: Severe fatigue determined by FSS mean score of 4 or higher; OR Severe pain determined by SF-36, bodily pain domain, score 55 or lower; OR Cognitive dysfunction defined by CFQ score 44 or higher. AND Limited physical activities determined by SF-36, physical function domain score 55 or lower
Other Comorbidities	No concurrent comorbidities that can explain illness.	

^a^ Criteria followed the 2006 IDSA case definition, with additional symptom confirmation by medical record review. Medical records consisted of details of current illness, standard-of-care physical exam findings, treatment and medical history including current and past LB diagnoses. Abbreviations: CFQ = Cognitive Failures Questionnaire; FSS = Fatigue Severity Scale; IDSA = Infectious Disease Society of America; LB = Lyme borreliosis; PTLDS = post-treatment Lyme disease syndrome; SF-36 = 36-Item Short Form Health Survey.

**Table 2:** Demographic and clinical characteristics of LB cases over Visits 3–5 and controls over Contacts 1–2.

Category	Enrolled Medically Attended LB Cases Who Completed Antibiotic Treatment with Resolution or Stabilization	Persistent Symptoms LB Cases	PTLDS Assessed by Questionnaires	PTLDS Assessed by Clinicians	Controls
	(*N* = 242)	(*N* = 22)	(*N* = 8)	(*N* = 11)	(*N* = 288)
Sex, *n* (%)					
Male	107 (44.2)	9 (40.9)	5 (62.5)	7 (63.6)	136 (47.2)
Female	135 (55.8)	13 (59.1)	3 (37.5)	4 (36.4)	152 (52.8)
Age at consent (Years)					
Mean (SD)	55.7 (15.66)	59.6 (13.61)	60.4 (13.53)	50.8 (14.23)	55.8 (15.74)
Min, Max	6, 86	31, 81	31, 74	31, 74	6, 86
*n* (%)					
5 to 17	5 (2.1)	0 (0.0)	0 (0.0)	0 (0.0)	6 (2.1)
18 to 34	17 (7.0)	1 (4.5)	1 (12.5)	1 (9.1)	22 (7.6)
35 to 64	139 (57.4)	12 (54.5)	3 (37.5)	7 (63.6)	164 (56.9)
≥65	81 (33.5)	9 (40.9)	4 (50.0)	3 (27.3)	96 (33.3)
History of LB, *n* (%)					
Yes	83 (34.3)	12 (54.5)	5 (62.5)	6 (54.5)	43 (14.9)
No	159 (65.7)	10 (45.5)	3 (37.5)	5 (45.5)	245 (85.1)
History of known tick bite(s), *n* (%)					
Yes	152 (62.8)	10 (45.5)	4 (50.0)	6 (54.5)	176 (61.1)
No	90 (37.2)	12 (54.5)	4 (50.0)	5 (45.5)	112 (38.9)
Highest level of education attained, *n* (%)					
Primary and Secondary School	155 (64.0)	16 (72.7)	6 (75.0)	6 (54.5)	203 (70.5)
University/Graduate School	83 (34.3)	6 (27.3)	2 (25.0)	5 (45.5)	84 (29.2)
Not Applicable ^a^	3 (1.2)	0 (0.0)	0 (0.0)	0 (0.0)	1 (0.3)
Missing	1 (0.4)	0 (0.0)	0 (0.0)	0 (0.0)	0 (0.0)
Occupation, *n* (%)					
Outdoor	39 (16.1)	4 (18.2)	2 (25.0)	2 (18.2)	39 (13.5)
Indoor	98 (40.5)	6 (27.3)	1 (12.5)	4 (36.4)	129 (44.8)
Student	4 (1.7)	0 (0.0)	0 (0.0)	0 (0.0)	6 (2.1)
No Occupation/Not Applicable ^b^	97 (40.1)	12 (54.5)	5 (62.5)	5 (45.5)	112 (38.9)
Missing or Unknown	4 (1.7)	0 (0.0)	0 (0.0)	0 (0.0)	2 (0.7)
Country, *n* (%)					
Czech Republic	26 (10.7)	3 (13.6)	0 (0.0)	1 (9.1)	31 (10.8)
Germany	20 (8.3)	1 (4.5)	0 (0.0)	1 (9.1)	24 (8.3)
Poland	8 (3.3)	1 (4.5)	0 (0.0)	0 (0.0)	7 (2.4)
Slovakia	89 (36.8)	14 (63.6)	7 (87.5)	6 (54.5)	106 (36.8)
Slovenia	16 (6.6)	0 (0.0)	0 (0.0)	0 (0.0)	21 (7.3)
Sweden	83 (34.3)	3 (13.6)	1 (12.5)	3 (27.3)	99 (34.4)
Disease manifestation, *n* (%)					
Localized	157 (64.9)	6 (27.3)	1 (12.5)	4 (36.4)	0 (0.0)
Disseminated ^c^	85 (35.1)	16 (72.7)	7 (87.5)	7 (63.6)	0 (0.0)
CCI Score					
Mean (SD)	2.8 (2.10)	3.5 (2.11)	3.9 (2.17)	2.1 (1.92)	2.5 (1.91)
Minimum, Maximum	0, 12	0, 8	0, 7	0, 7	0, 10

^a^ Persons who have no school education (including children <4 years). ^b^ Persons who do not have an occupation. ^c^ Disseminated manifestations among the “persistent symptoms” subgroup were Lyme neuroborreliosis (1), Lyme arthritis (12), and LB diagnoses without established LB clinical manifestations (3). All 7 disseminated PTLDS cases, as assessed by questionnaires, were Lyme arthritis. The 7 disseminated PTLDS cases as assessed by clinicians consisted of 1 Lyme neuroborreliosis, 4 Lyme arthritis, 1 LB diagnosis without established LB clinical manifestations (*Borrelia* positive blood results), and 1 with more than one manifestation (erythema migrans and Lyme arthritis). Abbreviations: CCI = Charlson Comorbidity Index; LB = Lyme borreliosis; PTLDS = post-treatment Lyme disease syndrome; SD = standard deviation.

**Table 3:** Antibiotic utilization to treat LB by subgroup over Visits 3–5.

Category	Enrolled Medically Attended LB Cases Who Completed Antibiotic Treatment with Resolution or Stabilization	Persistent Symptoms LB Cases	PTLDS Assessed by Questionnaires	PTLDS Assessed by Clinicians
	(*N* = 242)	(*N* = 22)	(*N* = 8)	(*N* = 11)
Number of different antibiotics used, *n* (%)				
0	0 (0.0)	0 (0.0)	0 (0.0)	0 (0.0)
1	228 (94.2)	20 (90.9)	7 (87.5)	8 (72.7)
2	13 (5.4)	1 (4.5)	0 (0.0)	2 (18.2)
>2	1 (0.4)	1 (4.5)	1 (12.5)	1 (9.1)
Total number of days using antibiotic(s) ^a^, *n* (%)				
0 days	0 (0.0)	0 (0.0)	0 (0.0)	0 (0.0)
1–6 days	5 (2.1)	1 (4.5)	0 (0.0)	1 (9.1)
7–14 days	136 (56.2)	9 (40.9)	5 (62.5)	4 (36.4)
15–21 days	59 (24.4)	4 (18.2)	1 (12.5)	2 (18.2)
22–28 days	24 (9.9)	5 (22.7)	1 (12.5)	2 (18.2)
>28 days	18 (7.4)	3 (13.6)	1 (12.5)	2 (18.2)
Total number of days using antibiotic(s) ^b^				
Mean	15.1	19.3	20.0	21.5
Median	11.0	18.0	10.0	18.0
Minimum, Maximum	2, 72	6, 72	9, 72	6, 72
Antibiotics used, *n* ^b^ (%)				
Doxycycline	85 (35.1)	8 (36.4)	1 (12.5)	2 (18.2)
Cefuroxime axetil	3 (1.2)	1 (4.5)	0 (0.0)	2 (18.2)
Amoxicillin	11 (4.5)	1 (4.5)	0 (0.0)	1 (9.1)
Ceftriaxone	2 (0.8)	1 (4.5)	0 (0.0)	1 (9.1)
Azithromycin	62 (25.6)	9 (40.9)	5 (62.5)	2 (18.2)
Clarithromycin	9 (3.7)	1 (4.5)	1 (12.5)	1 (9.1)
Penicillin G	0 (0.0)	0 (0.0)	0 (0.0)	0 (0.0)
Phenoxymethylpenicillin	70 (28.9)	1 (4.5)	1 (12.5)	2 (18.2)
When patient started antibiotic treatment, *n* ^b^ (%)				
Within 30 days of first documented symptom	190 (78.5)	11 (50.0)	3 (37.5)	6 (54.5)
More than 30 days after first documented symptom	52 (21.5)	11 (50.0)	5 (62.5)	5 (45.5)

^a^ Treatment duration on antibiotics was calculated based on known start and end dates, as collected in the case report form. ^b^ Among patients who completed at least one antibiotic for LB. Abbreviations: LB = Lyme borreliosis; PTLDS = post-treatment Lyme disease syndrome.

## Data Availability

The datasets used and/or analyzed during the current study are not publicly available due to privacy or ethical restrictions and are available from the corresponding author on reasonable request.

## Abbreviations

The following abbreviations are used in this manuscript:

BOLD	Burden of Lyme Disease
CCI	Charlson Comorbidity Index
CFQ	Cognitive Failures Questionnaire
CI	Confidence interval
CIS	Checklist Individual Strength
FSS	Fatigue Severity Scale
HRQoL	Health-related quality of life
LB	Lyme borreliosis
PRO	Patient-reported outcome
PTLDS	Post-treatment Lyme disease syndrome
SF-36	36-Item Short Form Health Survey.

## Appendix A

Investigators of the BOLD Study Group include Dr Lenka Dybova (Doktor Brno S.R.O., Brno, Czech Republic), Dr Miroslav Pavlasek (ADMED, S.R.O., Ceske Budejovice, Czech Republic), Dr Daniela Verdanova (Ordinace praktickeho lekare pro deti a dorost, Jindrichuv Hradec, Czech Republic), Dr Joachim Weimer (Private Practice of Dr. Joachim Weimer, Reinfeld, Germany), Dr Christine Kosch (Private Practice of Dr. Christine Kosch, Pirna, Germany), Dr Kai Mehltretter (Velocity Clinical Research, Leipzig, Germany), Dr. Hans-Detlev Stahl (AmBeNet GmbH, Leipzig, Germany), Dr Barbara Nieradko-Iwanicka (Lubelskie Centrum Diagnostyczne, Swidnik, Poland and Medical University of Lublin, Lublin, Poland), and Dr Dorota Bielska (Akademicka Praktyka Medycyny Rodzinnej; Bialystok, Poland). The BOLD Study Group also includes: Drs Berglund, Moniusko-Malinowska, Strle, Cibik, and Zakova, who are included in the co-authorship list.

## Appendix B

**Table A1:** Ethics committee approval information.

Country	Ethics Committee	Initial Approval Date	Initial Approval Code
Czech Republic	Eticka komise IKEM a FTN Fakultni Thomayerova nemocnice Videnska 800 Praha 4—Krc, 140 59	16 February 2021	12/21+4217/21
Germany	Ethik-Kommission bei der Landesärztekammer Hessen Hanauer Landstrasse 152 Frankfurt am Main, 60314	12 March 2021	2021-2414-zvBO
Germany	Ethikkommission bei der Ärztekammer Schleswig-Holstein Bismarckallee 8–12 Bad Segeberg, 23795	12 March 2021	034/21 m
Germany	Ethikkommission bei der Sächsischen Landesärztekammer Schutzenhohe 16 Dresden, SACHSEN 01099	12 March 2021	EK-BR-22/21-1
Poland	Komisja Bioetyczna przy Lubelskiej Izbie Lekarskiej w Lublinie ul. Chmielna 4 Lublin, 20-079	1 February 2021	140/2021/KB/VIII
Poland	Komisja Bioetyczna przy Uniwersytecie Medycznym w Bialymstoku ul. Jana Kilinskiego 1 Bialystok, 15-089	25 March 2021	APK.002.169.2021
Poland	Komisja Bioetyczna przy Okregowej Izbie Lekarskiej z siedziba w Bialymstoku ul. Swietojanska 7 Bialystok, 15-082	21 April 2021	12/2021/VIII
Slovakia	Eticka Komisia Trencianskeho samospravneho Karja K dolnej stanici 7282/20A Trencin, 911 01	10 February 2021	No code provided—the approval references Pfizer’s protocol number: C4601002
Slovenia	Komisija Republike Slovenije za medicinsko etiko Stefanova 5 Ljubljana, 1000	31 May 2021	0120-53/2021-4
Sweden	Etikprövningsmyndigheten Box 2110 Uppsala, 750 02	5 May 2021	2021-00604

## Appendix C

**Table A2:** Frequency of enrolled medically attended LB cases with PTLDS by manifestation and definition.

Patient Met Criteria for PTLDS at Month 10 or 18	Satisfy Either PTLDS Definitions *N* (%)	PTLDS Assessed by Clinicians *N* (%) ^a^	PTLDS Assessed by Questionnaires *N* (%)	Satisfy Both Definitions *N* (%)
Localized	4 (26.7)	4 (36.4)	1 (12.5)	1 (25.0)
Disseminated	11 (73.3)	7 (63.6)	7 (87.5)	3 (75.0)
LB cases	15 (100.0)	11 (100.0)	8 (100.0)	4 (100.0)

^a^ Of the 11 patients who were assessed by clinicians for PTLDS, 10 were assessed at month 10, and 1 additional patient with localized LB was assessed at month 18. Abbreviations: LB = Lyme borreliosis; PTLDS = post-treatment Lyme disease syndrome.

**Table A3:** Number of enrolled medically attended LB with persistent symptoms or PTLDS cases who satisfied individual PRO criteria.

	Persistent Symptom Cases (*N* = 22)	PTLDS Cases Assessed by Questionnaires (*N* = 8)	PTLDS Cases Assessed by Clinicians (*N* = 11) ^a^
Cases defined by SF-36 Bodily Pain subscale ≤55, *n* (%)	20 (90.9)	8 (100.0)	7 (63.6)
Cases defined by CFQ score ≥44, *n* (%)	9 (40.9)	3 (37.5)	2 (18.2)
Cases defined by FSS score ≥4, *n* (%)	18 (81.8)	8 (100.0)	7 (63.6)
Cases defined by SF-36 Physical Function subscale ≤55, *n* (%)	9 (40.9)	8 (100.0)	4 (36.4)

^a^ Of the 11 patients who were clinically assessed for PTLDS, 10 were assessed at month 10, and 1 additional patient was assessed at month 18. Abbreviations: CFQ = Cognitive Failures Questionnaire; FSS = Fatigue Severity Scale; LB = Lyme borreliosis; PRO = patient-reported outcome; PTLDS = post-treatment Lyme disease syndrome; SF-36 = 36-Item Short Form Health Survey.
